# Opposite and Differently Altered Postmortem Changes in H3 and H3K9me3 Patterns in the Rat Frontal Cortex and Hippocampus

**DOI:** 10.3390/epigenomes8010011

**Published:** 2024-03-18

**Authors:** Karolina Dulka, Noémi Lajkó, Kálmán Nacsa, Karoly Gulya

**Affiliations:** Department of Cell Biology and Molecular Medicine, University of Szeged, 6720 Szeged, Hungary; dulka.karolina@med.u-szeged.hu (K.D.); lajko.noemi@med.u-szeged.hu (N.L.); kalman.nacsa@ek.szte.hu (K.N.)

**Keywords:** epigenetics, frontal cortex, H3, H3K9me3, hippocampus, postmortem delay

## Abstract

Temporal and spatial epigenetic modifications in the brain occur during ontogenetic development, pathophysiological disorders, and aging. When epigenetic marks, such as histone methylations, in brain autopsies or biopsy samples are studied, it is critical to understand their postmortem/surgical stability. For this study, the frontal cortex and hippocampus of adult rats were removed immediately (controls) or after a postmortem delay of 15, 30, 60, 90, 120, or 150 min. The patterns of unmodified H3 and its trimethylated form H3K9me3 were analyzed in frozen samples for Western blot analysis and in formalin-fixed tissues embedded in paraffin for confocal microscopy. We found that both the unmodified H3 and H3K9me3 showed time-dependent but opposite changes and were altered differently in the frontal cortex and hippocampus with respect to postmortem delay. In the frontal cortex, the H3K9me3 marks increased approximately 450% with a slow parallel 20% decrease in the unmodified H3 histones after 150 min. In the hippocampus, the change was opposite, since H3K9me3 marks decreased steadily by approximately 65% after 150 min with a concomitant rapid increase of 20–25% in H3 histones at the same time. Confocal microscopy located H3K9me3 marks in the heterochromatic regions of the nuclei of all major cell types in the control brains: oligodendrocytes, astrocytes, neurons, and microglia. Therefore, epigenetic marks could be affected differently by postmortem delay in different parts of the brain.

## 1. Introduction

Epigenetics is the study of mitotically heritable, but reversible, changes in gene expression that occur without a change in the genomic DNA sequence [1,2]. Epigenetic modifications are critical in neural development [3,4,5], aging [6,7], and neurological disorders [8,9,10,11,12]. Although animal models have provided valuable information on epigenetic events in the brain [13], the study of human brain tissue is necessary to fully understand human pathologies. Because it is not yet feasible to perform in vivo epigenetic studies for tissues such as the brain, only retrospective study designs using postmortem brain samples are viable [14]. Variations in circumstances before death or postmortem delay can influence gene expression and molecular constituents of brain tissue [14,15,16,17,18,19,20]. For human autopsies, postmortem delays from several hours up to a day are typical [21,22], while much shorter delays between experimental intervention and tissue preservation can be measured in animal models. For example, high levels of acetylation and methylation at certain sites of the H3 and H4 core histones in liver cells were found to be evident within 60 min of growth hormone treatment [23,24]. Therefore, it is important to understand postmortem changes in epigenetic modifications and report postmortem delay along with other vital parameters of the patients [21,25,26].

Epigenetic mechanisms act in advance of specific gene transcription events and are encoded in various histone post-translational modification (PTM) patterns, as well as in DNA methylation [18,19,27]. One of the main epigenetic modifications is histone methylation [2]. Reversible addition and removal of the methyl group(s) in the amino group (mono-, di-, or trimethylation) is governed by histone methyltransferases and histone demethylases [28]. In particular, in the case of histone H3, its PTMs are closely related to fundamental cellular events such as activation and repression of transcription. Methylation of histone lysine (K) residues plays an important and varied role in the regulation of gene expression [29]. The histone H3 methylation marks that are linked to transcriptional activation are H3K4, H3K36, and H3K79, while the transcriptional repression-specific methylation marks are H3K9 and H3K27 [30]. However, one methyl mark itself might have only a limited biological relevance. H3K9 trimethylations have been well studied due to their fundamental roles in heterochromatin formation, transcriptional silencing, X chromosome inactivation, and DNA methylation [31]. These modifications are associated with changes in gene transcription through alteration of the chromatin structure [32]. For example, H3K9me3 is a typical mark of constitutive heterochromatin [33].

We hypothesized that postmortem delay (the time between death and tissue removal/preservation for study) would affect epigenetic modifications in brain tissue. We addressed this by determining the stability of histone methylation in samples from different areas of the brain after death using Western blot analysis and confocal immunohistochemistry. In the present study, we examined the H3 and H3K9me3 patterns as a function of postmortem delay in two distinct regions of the brain of the adult rat.

## 2. Results

**Figure 1:**
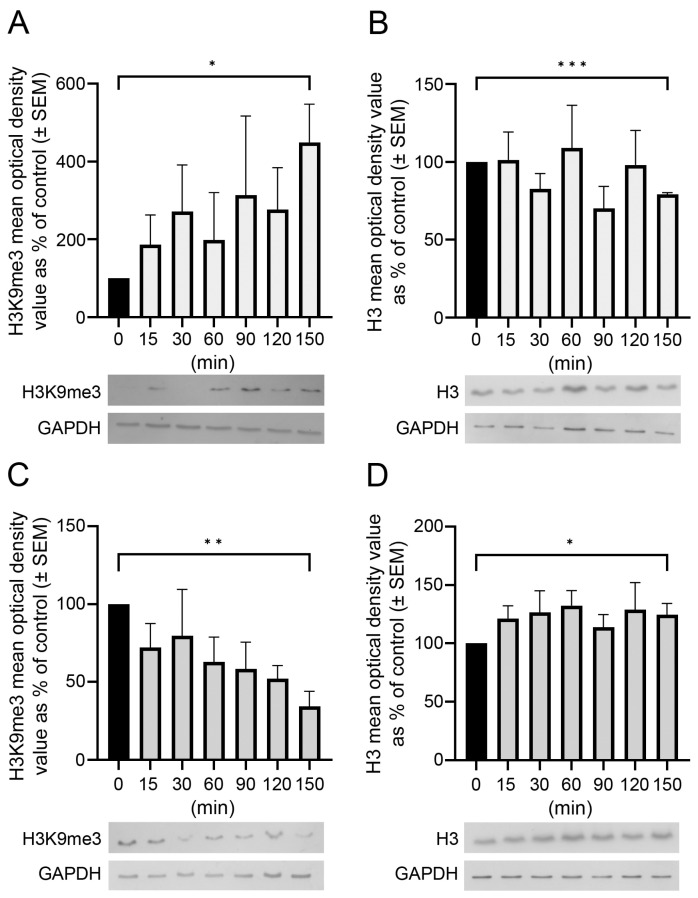
Postmortem delay-dependent changes in H3 and H3K9me3 immunoreactivities in the frontal cortex and hippocampus of rats. Quantitative Western blot analysis on samples from the frontal cortex (**A**,**B**) and hippocampus (**C**,**D**) was performed as described in Section 4. Samples were analyzed from control (delayed by 0 min) and postmortem-delayed (by 15–150 min) brain tissues. Three and four sets of animals were used for studying histone levels in the hippocampus and frontal cortex, respectively. The immunoreactive densities of equally loaded lanes on the membranes were quantified and the samples were normalized to internal (GAPDH) controls. Data are presented as mean % of control ± SEM from three to four separate experiments. Representative Western blots are shown below the graphs. Statistical analyses were performed using GraphPad Prism for Windows (v. 9.4.0). Differences were calculated using an unpaired two-tailed Student’s *t*-test. Asterisks denote statistical significances: * *p* < 0.05; ** *p* < 0.01, *** *p* < 0.001.

The postmortem delay progressively affected the amounts and patterns of immunologically detectable H3 and H3K9me3. Western blot analysis showed that changes in the amounts of unmodified H3 and H3K9me3 immunosignals were time-dependent and altered oppositely in the frontal cortex and hippocampus with respect to postmortem delay (Figure 1). For example, H3K9me3 marks increased by about 450% (* *p* < 0.05 at 150 min; Figure 1A), with a parallel slow 20% decrease in unmodified H3 histones (*** *p* < 0.001 at 150 min) in the frontal cortex (Figure 1B). However, in the hippocampus, the change was opposite, as the H3K9me3 marks decreased steadily by about 65% (** *p* < 0.01) after a postmortem delay of 150 min (Figure 1C), with a concomitant increase of 20–25% in the H3 histones (* *p* < 0.05) at the same time (Figure 1D).

**Figure 2:**
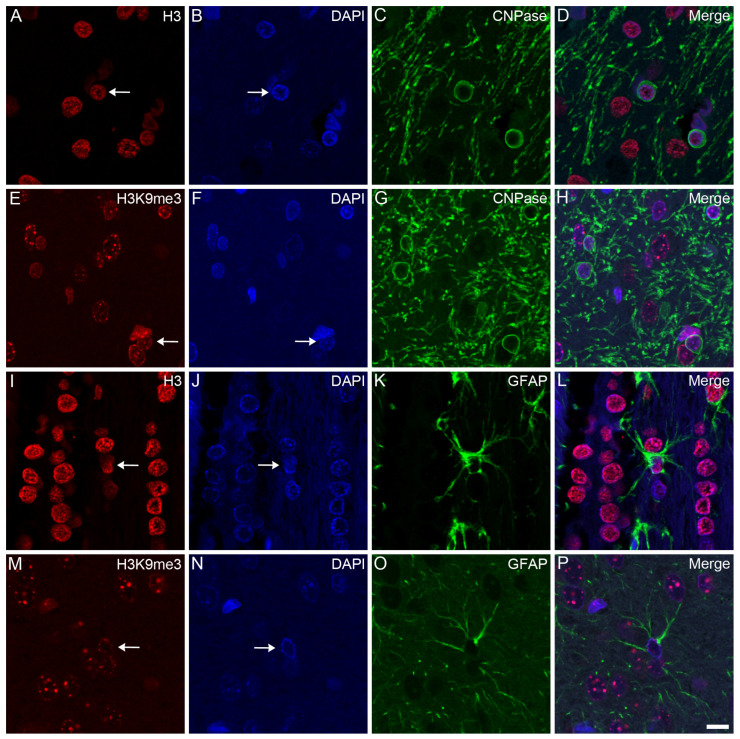
Immunohistochemical localization of H3 and H3K9me3 in oligodendrocytes and astrocytes in the frontal cortex of control rats. The immunolabeled sections were examined with a confocal laser scanning microscope, as described in Section 4. Oligodendrocytes and astrocytes were labeled with anti-CNPase (**C**,**G**) and anti-GFAP (**K**,**O**) antibodies, respectively. The tissue sections were also stained with an anti-H3 (**A**,**I**) or anti-H3K9me3 (**E**,**M**) antibody and counterstained with DAPI (**B**,**F**,**J**,**N**). The merged images (**D**,**H**,**L**,**P**) show the distributions of H3 (**D**,**L**) and H3K9me3 (**H**,**P**) relative to the heterochromatic foci labeled with DAPI. H3 and H3K9me3 distributed diffusely in oligodendrocytes (arrows in (**A**,**E**)) and astrocytes (arrows in (**I**,**M**)), where the immunolabel was accompanied by a typically marginal heterochromatin (arrows in (**B**,**F**,**J**,**N**)). Scale bar in (**P**): 10 μm.

**Figure 3:**
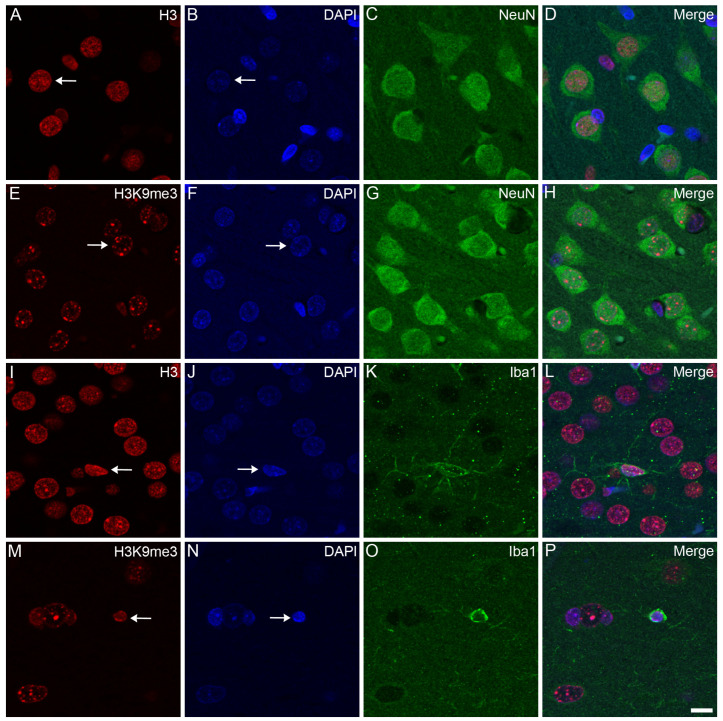
Immunohistochemical localization of H3 and H3K9me3 in neurons and microglia in the frontal cortex of control rats. The immunolabeled sections were examined with a confocal laser scanning microscope, as described in Section 4. Neurons and microglia were labeled with anti-NeuN (**C**,**G**) and anti-Iba1 (**K**,**O**) antibodies, respectively. The tissue sections were also stained with an anti-H3 (**A**,**I**) or anti-H3K9me3 (**E**,**M**) antibody and counterstained with DAPI (**B**,**F**,**J**,**N**). The merged images (**D**,**H**,**L**,**P**) show the distributions of H3 (**D**,**L**) and H3K9me3 (**H**,**P**) relative to the heterochromatic foci labeled with DAPI. Note that while the immunolabel for H3 in the neurons (arrow in (**A**)) and microglia (arrow in (**I**)) is diffuse, the signal for H3K9me3 in the neurons (arrow in (**E**)) and microglia (arrow in (**M**)) is more puncta-like. While the chromatin shows weak DAPI staining in the neurons (arrows in (**B**,**F**)), its stains are stronger and more heterochromatic in the microglia (arrows in (**J**,**N**)). Scale bar in (**P**): 10 μm.

**Figure 4:**
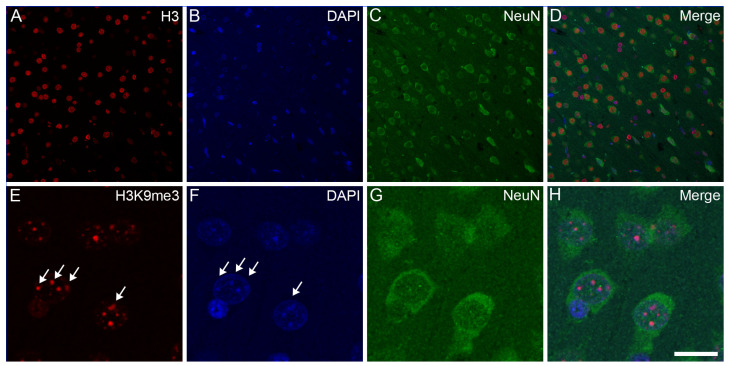
Subnuclear localization of H3 and H3K9me3 immunosignals in neurons in the frontal cortex of control rats. The immunolabeled sections were examined with a confocal laser scanning microscope, as described in Section 4. The neurons were labeled with an anti-NeuN antibody (**C**,**G**) together with either an anti-H3 (**A**) or anti-H3K9me3 (**E**) antibody, and counterstained with DAPI (**B**,**F**). The merged images (**D**,**H**) show H3 and H3K9me3 distributions, respectively, relative to DAPI-labeled heterochromatic foci. Several intense clusters of H3K9me3 marks (arrows in (**E**)) are visible in most neuronal nuclei (**H**). Note that H3K9me3 immunoreactivity in the NeuN-labeled neurons is localized predominantly to transcriptionally inactive heterochromatin that overlap with DAPI-labeled heterochromatin (arrows in (**E**,**F**)). Scale bar in (**H**): 20 μm.

Confocal microscopy localized the immunosignals of unmodified H3 and K3K9me3 primarily in heterochromatic regions, labeled with the fluorescent DNA intercalator DAPI, in all major cell types in the frontal cortex (Figure 2, Figure 3 and Figure 4) and hippocampus (data not shown) of the control animals: oligodendrocytes (Figure 2A–H), astrocytes (Figure 2I–P), neurons (Figure 3A–H), and microglia (Figure 3I–P). Similarly, for all major cell types analyzed in the frontal cortex, the nuclear distribution of unmodified H3 was more homogeneous (Figure 4A–D), while the H3K9me3 immunolabel was clustered as puncta (arrows in Figure 4E) and was predominantly found at the chromocenters of the neuronal nuclei (Figure 4E–H). The intensity of the DAPI stain was typically localized in a few dense chromocenters and in the marginal heterochromatin (arrows in Figure 4F). Based on NeuN immunofluorescence, the majority of cells were neurons in the adult brain tissue (Figure 3D–H and Figure 4D–H).

## 3. Discussion

Postmortem brain tissue is invaluable for the study of disorders of the human nervous system. After autopsy, brain samples must be studied immediately or preserved for later diagnosis that could include molecular biological/proteomic work. However, premortem and postmortem factors can interfere with molecular biology studies, and erroneous conclusions might be drawn because of the disregard of possible sources of pitfalls and artifacts [16,34]. For example, a recent study showed that even a brief (20 min) general anesthesia promoted the hypermethylation of certain genes in the brain [35]. Postmortem variables include the environment after death, storage temperature, time between death and autopsy (postmortem delay), and preservation method [34].

There are several studies that aim to study ultrastructural, biochemical, molecular biological, gene expression, etc., changes in the nervous system as a function of time after death. For example, a number of in vivo and in vitro studies have examined ultrastructural changes in organelles such as the mitochondria and endoplasmic reticulum [36,37,38,39]. In many cases, significant changes could be detected as a consequence of the hypoxic tissue environment [38,40,41,42]. For example, rats kept under mild hypoxic conditions for a short period of time (16% O_2_ environment for 60 min) exhibited an increase in certain interneuron populations showing early signs of neurodegeneration, detected by Gallyas-type silver impregnation [43], in different parts and layers of the hippocampus compared to normoxic conditions (21% O_2_) [40]. Most of the compacted silver-impregnated inhibitory neurons were also immunopositive for somatostatin. Ultrastructural changes associated with such silver-impregnated dark neurons as early forms of neurodegeneration have been described in detail [43].

In another study, neurohypophyseal pituicytes and neurosecretory axons were ultrastructurally examined after experimentally induced clinical death lasting 15 min [39]. Hyperactive forms of pituicytes rich in subcellular organelles, including lysosomes, as well as hypoactive forms of cells scarce in organelles but containing numerous electron-lucent vacuoles were observed in neurohypophysis after the hypoxic period. Hypoxia alters not only cellular characteristics but also biochemical characteristics. For example, hypobaric (mimicking an altitude of 9000 m, where air pressure = 30.7 kPa, pO_2_ = 6.4 kPa), short-term hypoxia (30 min) increased lipid peroxide formation in different parts of developing and adult rat brains [44].

In vitro studies are also used to study hypoxic or anoxic conditions. For instance, an electron microscopic examination of organotypic cultures of the rat hippocampus, exposed to 10 and 20 min anoxic insults, revealed some morphological characteristics typical of both necrotic and apoptotic neuronal cell death: for example, organelle swelling and nuclear chromatin condensation, respectively [41]. In another study, using primary microglial cell culture (BV-2), short-term hypoxia (30 min) significantly increased the expression of a subset of pro-inflammatory (tumor necrosis factor α, interleukin 1β) and oxidative stress-related (hypoxia-inducible factor 1α) genes [45].

In one human study, the astroglial calcium-binding protein S-100, an early and sensitive marker of hypoxic brain damage and outcome of cardiac arrest in humans, was studied [46]. In 66 patients who underwent cardiopulmonary resuscitation after non-traumatic cardiac arrest, blood samples were drawn immediately after; 15, 30, 45, and 60 min after; 2, 8, 24, 48, and 72 h after; and 7 days after the initiation of cardiopulmonary resuscitation. They found that maximum levels of S-100 within 2 h after cardiac arrest were significantly higher in patients with documented brain damage than in patients without brain damage. Furthermore, significant differences between these two groups were observed from 30 min to 7 days after cardiac arrest. This study concluded that S-100 was a reliable early marker of brain damage and an outcome of hypoxia caused by cardiac arrest [46].

Postmortem delay also affects epigenetic modifications [14]. Accordingly, Jarmasz et al. [19] showed that a postmortem delay of ≤24 h in brain tissue from pigs and mice had adverse effects on some histone acetylation marks; when several histone acetylation marks were tested in a human brain microarray, they were found to be relatively stable for up to 96 h. Therefore, special care must be taken to consider these circumstances when dealing with postmortem brain tissue from humans for research, as not all samples from such tissues are equally suitable for molecular biology studies.

Some epigenetic markers are associated with brain dysfunction and therefore could be important diagnostic markers. For example, one study found that a histone methyltransferase inhibitor decreased hippocampal H3K9me3, a site of lysine trimethylation, which is commonly involved in gene silencing, concomitantly improving performance in a complex spatial environment learning task in aged mice [47]. However, abnormal heterochromatin remodeling by H3K9me3 was recently shown to lead to down-regulation of genes related to synaptic function, suggesting that epigenetic alteration by H3K9me3 is associated with synaptic pathology seen in the sporadic form of Alzheimer’s disease [48]. H3K9me3 levels are negatively correlated with memory, since high levels have been measured in the hippocampus of old male mice; a varying pattern of H3K9me3 expression was also observed in different subregions of the aging hippocampus [49]. Furthermore, while they were increased by acute stress, chronic stress decreased H3K9me3 patterns in the dentate gyrus and CA1 region of the hippocampus, indicating a relatively rapid regionally specific change in methylation mechanisms prior to death [50]. Therefore, not only age-related but stress-related differences could influence neuronal epigenetic mark levels. However, the possible diagnostic value of these epigenetic alterations could rapidly decrease when there is a substantial postmortem delay.

We demonstrated a brain region-dependent distribution of H3K9me3 marks that change as a function of postmortem delay. We also showed that as the H3K9me3 marks changed, unmodified H3 patterns reciprocally changed in both tissues in a time-dependent manner: as H3K9me3 marks increased, unmodified H3 histones decreased in the frontal cortex, while the opposite direction of change was observed in the hippocampus. As the degree of conversion between histone types was different, we speculate that this could be due to different activities of epigenetic readers, writers, and erasers in different subregions of the brain. Most H3K9me3 marks were in heterochromatic regions of the nucleus, consistent with previous studies that placed this immunolabel on late-replicating pericentric heterochromatin in mouse cells and at sites of DAPI-dense intranuclear heterochromatin in human and hamster cells [51]. This intranuclear localization supports the inhibitory role that these marks play during transcription. Neurons are the dominant cell type in the adult rat brain, comprising about 60% of the total number of cells [52]. Therefore, due to their abundance, they probably contribute the most to the postmortem delay-related distribution of H3 and H3K9me3 seen in the rat frontal cortex and hippocampus.

Histone demethylases are epigenetic erasers that remove methyl groups from modified histones, thus activating or suppressing gene transcription [53,54]. Histone demethylases such as the Jumonji domain-containing protein 2B (JMJD2B) or lysine-specific demethylase 1 (LSD1) remove methylation marks on lysine 9 in histone H3 (H3K9me2/3, H3K4me1/2, or H3K9me1/2) and work as transcription activators [55,56]. We theorize that histone demethylase activity is still preserved and enzymes such as JMJD2B and LSD1 are still capable of converting trimethylated, dimethylated, and monomethylated H3K9 marks during a prolonged postmortem delay. In fact, Lu et al. [57] demonstrated that hypoxic stress decreased H3K4 methylation, another target of LSD1 [58]; hypoxia is often present around the time of death and could therefore precipitate the demethylation of lysine sites, followed by the accumulation of unmodified H3. It should be noted that (1) the antibody we used to detect H3K9me3 did not recognize mono- or dimethylated forms at this site, and (2) we did not have antibodies to detect mono- and dimethylated forms of H3K9; so, we were unable to quantitatively analyze how different demethylation processes increased the amount of unmodified H3 during postmortem delay.

In summary, we demonstrated time-dependent and opposite alterations of histone patterns in two regions of the brain as functions of postmortem delay. When evaluating potential chromatin stress responses, thorough documentation of all possible regulatory effects on epigenetic marks, such as vital statistics, premortem clinical diagnoses (trauma, medication, infection, inflammation, stress circumstances, substance abuse, etc.), as well as postmortem delay and autopsy findings, should be available. We believe that our results contribute to a better understanding of the stability of epigenetic marks and may have diagnostic implications. Furthermore, our results show the importance of postmortem delay in analyzing gene regulatory factors, such as post-translational histone modifications.

## 4. Materials and Methods

### 4.1. Animal Handling and Establishment of Postmortem Delay

We carried out all animal experiments in strict compliance with the European Council Directive (86/609/EEC) and the EC regulations (O.J. of EC No. L 358/1, 18/12/1986) regarding the care and use of laboratory animals for experimental procedures and followed the relevant requirements of Hungarian and local legislation. The experimental protocols presented in this study were approved by the Institutional Animal Welfare Committee of the University of Szeged (II./1131/2018). Adult female Sprague Dawley rats (190–210 g) were kept in standard housing conditions and fed ad libitum with regular laboratory food. After the animals were decapitated and the heads were kept at room temperature (RT) for different time periods to provide different postmortem delays (0, 15, 30, 60, 90, 120, and 150 min), the brains were removed and the frontal cortex and hippocampus were dissected for further analysis. For Western blot analysis, 3 sets of animals that each underwent different postmortem delays were used for the study of hippocampal histone H3 and H3K9me3 levels, while 4 sets of rats were used for the analysis of H3 and H3K9me3 levels in the frontal cortex. For fluorescent immunohistochemistry, tissue samples from the frontal cortices of control animals were processed to provide representative light microscopic images to demonstrate the tissue cytoarchitecture and immunohistochemical distribution of the cell-specific and histone immunolabels.

### 4.2. Histology

The brains were removed, fixed via immersion in 0.05 M phosphate-buffered saline (PBS; pH 7.4 at 4 °C) containing 4% formaldehyde, and then embedded in paraffin for immunohistochemistry. Paraffin-embedded sections (6 µm thick) were cut on a microtome (Leica RM2235; Leica Mikrosysteme Vertrieb GmbH, Wetzlar, Germany), mounted on glass microscope slides coated with (3-aminopropyl)triethoxysilane (Menzel, Darmstadt, Germany), and used for confocal immunohistochemistry.

### 4.3. Antibodies

The primary and secondary antibodies used for the confocal immunohistochemistry and Western blot analyses are listed in Table 1. We used antibodies for cell-specific markers to detect neurons (anti-neuronal nuclei protein; anti-NeuN), astrocytes (anti-glial fibrillary acidic protein; anti-GFAP), oligodendrocytes (anti-2′,3′-cyclic nucleotide 3′-phosphodiesterase; anti-CNPase), and microglia (anti-ionized calcium-binding adaptor molecule 1; anti-Iba1). Furthermore, we used antibodies against the unmodified core histone H3 protein and its PTM at a lysine site, H3K9me3. An antibody raised against the enzyme glyceraldehyde 3-phosphate dehydrogenase (anti-GAPDH) was used as an internal control in the Western blot experiments.

### 4.4. Western Blot Analysis

Brain samples (frontal cortex and hippocampus) from the adult rats were dissected at each time point, homogenized in a 50 mM Tris–HCl (pH 7.5 at 4 °C) solution containing 150 mM NaCl, 1 μg/mL pepstatin, 2 μg/mL leupeptin, 2 mM phenylmethylsulfonyl fluoride, and 2 mM EDTA. The homogenate was centrifuged at 10,000× *g* for 10 min. The supernatant was aliquoted and the pellet was homogenized in a 50 mM Tris–HCl (pH 7.5 at 4 °C) solution containing 150 mM NaCl, 0.1% Nonidet P40, 0.1% cholic acid, 1 μg/mL pepstatin, 2 μg/mL leupeptin, 2 mM phenylmethylsulfonyl fluoride, and 2 mM EDTA. Protein concentration was determined using the method of Lowry et al. [59]. For Western blot analysis, 10–15 μg of proteins was separated on a sodium dodecyl sulfate/polyacrylamide gel (4–12% stacking gel/resolving gel). After being separated, the proteins were transferred to Hybond-ECL nitrocellulose membranes (Amersham Biosciences, Little Chalfont, Buckinghamshire, England), the membranes were blocked for 1 h in 5% non-fat dry milk in Tris-buffered saline (TBS) containing 0.1% Tween-20 (to prevent nonspecific antibody binding), and incubated overnight with one of the appropriate primary antibodies (Table 1), as well as with that of the internal control (mouse anti-GAPDH monoclonal antibody). The membranes were subjected to 5 rinses in 0.1% TBS–Tween-20, then incubated for 1 h with secondary antibodies conjugated to peroxidase, and washed three times as above. We used the enhanced chemiluminescence method (ECL Plus Western blot detection reagents; Amersham Biosciences) to visualize immunoreactive bands according to the manufacturer’s protocol. The exposure time and development were optimized for each antibody.

### 4.5. Confocal Immunohistochemistry

Immunohistochemistry was performed as previously described [13]. Paraffin-embedded tissue sections were deparaffinized, rehydrated, and placed in a Coplin jar filled with 0.01 M citrate buffer (pH 6.0) containing 0.05% Tween-20 and then heated at 95 °C for 20 min. The tissue sections were washed three times for 10 min, each in 0.05 M PBS solution containing 0.05% Tween-20. To prevent nonspecific antibody binding to the tissue, the sections were blocked in a 0.05 M PBS solution containing 0.05% Tween-20 and 5% normal goat serum (NGS) for 1 h at RT. The tissue sections were then incubated with primary antibodies in a 0.05 M PBS solution containing 0.05% Tween-20 and 5% NGS overnight at 4 °C. After extensive washing (4 × 10 min in 0.05 M PBS containing 0.05% Tween-20), the primary antibodies were labeled with secondary antibodies conjugated to one of the photostable fluorescent dyes (Alexa 488 or Alexa 568; Invitrogen, Carlsbad, CA, USA) in a blocking solution for 3 h at RT (for final dilutions, see Table 1). After 4 × 10 min rinses in 0.05 M PBS containing 0.05% Tween-20, cell nuclei were stained with a 2-[4-(aminoiminomethyl)phenyl]-1H-indole-6-carboximidamide hydrochloride solution (DAPI; ThermoFisher Scientific, Waltham, MA, USA). Immunolabeled tissue sections were examined with a Leica Stellaris DLS laser-scanning super-resolution confocal microscope (Leica Microsystems, Wetzlar, Germany). Digital images (1024 × 1024 pixels) were captured using the following confocal microscope configuration: objective lens, UPLSAPO 60×; numerical aperture, 1.35; sampling speed, 4 μs/pixel; optical zoom, 2×; scanning mode, sequential unidirectional. The excitation wavelengths were as follows: 405 nm (for DAPI), 488 nm (for Alexa Fluor 488), and 543 nm (for Alexa Fluor 568).

### 4.6. Image and Statistical Analyses

Digital grayscale images of the Western blots were acquired by scanning autoradiographic films with a 24-bit desktop scanner (Epson Perfection V750 PRO; Seiko Epson Corp., Suwa, Nagano, Japan). The scanned images were captured at a resolution of 600 × 600 dots per inch and processed in identical settings to allow comparisons of the Western blots of different samples. Bands were analyzed using the computer program ImageJ (version 1.47; developed at the US National Institutes of Health [60], available at https://imagej.net/Downloads, accessed on 10 July 2013). The mean values of the equally loaded lanes were quantified as follows: the background was subtracted, the data were normalized to the internal GAPDH load controls, and the data were expressed as a % of the control values. All statistical analyses were performed with GraphPad Prism for Windows software (version 9.4.0; GraphPad Software, San Diego, CA, USA). Data were presented as mean % of control ± standard error of the mean (SEM) of at least three immunoblots, each representing an independent experiment. Statistical differences were calculated using an unpaired two-tailed Student’s *t*-test for the Western blots. A *p* value of <0.05 was considered significant.

## Figures and Tables

**Table 1:** Antibodies used in Western blot analysis and confocal immunohistochemistry *.

Primary Antibody, Abbrev. Name	Primary Antibody, Full Name (Cat. No.)	Final Dilution	Company Name	Secondary Antibody with Fluorochrome, Full Name (Cat. No.)	Company Name	Final Dilution
NeuN	Mouse anti-NeuN, monocl. ab. (MAB377)	1:100	Chemicon, Temecula, CA, USA	Alexa Fluor 488 goat anti-mouse IgG (A-10680)	Invitrogen, Carlsbad, CA, USA	1:1000
GFAP	Mouse anti-GFAP, monocl. ab. (MA1-19395)	1:100	ThermoFisher Scientific, Inc., Waltham, MA, USA	Alexa Fluor 488 goat anti-mouse IgG (A-10680)	Invitrogen, Carlsbad, CA, USA	1:1000
CNPase	Mouse anti-CNPase, monocl. ab. (ab6319)	1:500	Abcam, Cambridge, UK	Alexa Fluor 488 goat anti-mouse IgG (A-10680)	Invitrogen, Carlsbad, CA, USA	1:1000
Iba1	Mouse anti-Iba1, monocl. ab. (016-26721)	1:500	FUJIFILM Wako Chemicals Europe GmbH, Neuss, Germany	Alexa Fluor 488 goat anti-mouse IgG (A-10680)	Invitrogen, Carlsbad, CA, USA	1:1000
H3	Rabbit anti-histone H3, polycl. ab., Chip Grade (ab1891)	1:1500	Abcam, Cambridge, UK	Alexa Fluor 488 goat anti-rabbit IgG (SAB4600389); Anti-rabbit IgG, perox. conjug. (WB) (A9169)	Sigma, St. Louis, MO, USA; Invitrogen, Carlsbad, CA, USA	1:2000; 1:1000 (WB)
H3K9me3	Rabbit anti-histone H3 (trimethyl K9), polycl. ab., Chip Grade (ab8898)	1:1000	Abcam, Cambridge, UK	Alexa Fluor 488 goat anti-rabbit IgG (SAB4600389); Anti-rabbit IgG, peroxidase conjug. (WB) (A9169)	Sigma, St. Louis, MO, USA; Invitrogen, Carlsbad, CA, USA	1:2000; 1:1000 (WB)
GAPDH	Mouse anti-GAPDH, monocl. ab., clone GAPDH-71.1 (G8795)	1:20,000	Sigma, St. Louis, MO, USA	Anti-mouse IgG, peroxidase conjug. (WB) (A9044)	Sigma, St. Louis, MO, USA	1:2000 (WB)

* Unless otherwise noted, final dilutions of primary and secondary antibodies are the same for both the immunohistochemistry and Western blot (WB) analyses. (WB): secondary antibody and final dilution for Western blot.

## Data Availability

All data are contained within the article.

## Abbreviations

CNPase	2′,3′-cyclic nucleotide 3′-phosphodiesterase (EC 3.1.4.37)
DAPI	2-[4-(aminoiminomethyl)phenyl]-1H-indole-6-carboximidamide hydrochloride
GAPDH	glyceraldehyde 3-phosphate dehydrogenase (EC 1.2.1.12)
GFAP	glial fibrillary acidic protein
H	histone
H3K	histone Lys modification
Iba1	ionized calcium-binding adaptor molecule 1
IgG	immunoglobulin G
NeuN	neuronal nuclei protein (hexaribonucleotide-binding protein-3
PBS	phosphate-buffered saline
PTM	post-translational modification
RT	room temperature
SEM	standard error of the mean
TBS	Tris-buffered saline
WB	Western blot
